# 50% efficacy dose of intravenous lidocaine in supressing sufentanil-induced cough in children: a randomised controlled trial

**DOI:** 10.1186/s12871-024-02541-6

**Published:** 2024-04-19

**Authors:** Yang Hu, Ming-cheng Du, Guo-hong Zhu, Xiang Long, Jing-jing Jiang, Yuan Gong

**Affiliations:** Institute of Anesthesiology and Critical Care Medicine, Three Gorges University & Yichang Central People’s Hospital, Yichang City, Hubei Province 443000 China

**Keywords:** Lidocaine, Anaesthesia, Sufentanil-induced cough, Paediatric, Opioids

## Abstract

**Background:**

Opioids such as sufentanil are used as anaesthetics due to their rapid action and superior analgesic effect. However, sufentanil induces a huge cough in paediatric patients. In contrast, intravenous (IV) lidocaine suppresses opioid-induced cough in children, but its use is limited due to anaesthetists’ concern about its toxicity. Therefore, this study aimed to evaluate the effect of dose-dependent IV lidocaine on sufentanil-induced cough (SIC) in paediatric patients.

**Methods:**

A total of 188 patients aged 3–12 years scheduled for elective tonsillectomy with or without adenoidectomy were enrolled and divided into four groups depending on different dose of lidocaine: A (0 mg.kg-1), B (1 mg.kg-1), C (1.5 mg.kg-1), and D (2 mg.kg-1). The primary outcome was the SIC grade observed during the induction of general anaesthesia. The secondary outcomes were the incidence of SIC, mean arterial pressure, and heart rate at T0, T1, T2, T3, T4, and T5.

**Results:**

The SIC grade was significantly different between groups A and D (*P* = 0.04) and between groups B and D (*P* = 0.03). Moreover, the incidence of SIC in groups A, B, C, and D was 81%, 87%, 68%, and 64%, respectively, and the difference between groups B and C (*P* = 0.03) and between groups B and D (*P* = 0.0083) was statistically significant. No statistical differences were observed in the hemodynamic parameters between the groups. The incidence of severe cough was statistically different between group D and group A (*P* < 0.0001), between group D and group B (*P* < 0.0001), and between group D and group C (*P* < 0.0001) respectively.

**Conclusions:**

Lidocaine suppresses SIC in a dose-dependent manner without severe adverse events. IV lidocaine can be used in paediatric patients safely and efficiently, and the median effective dose was 1.75 mg/kg.

**Trial registration:**

This study was approved by the Institutional Review Board of Yichang Central People’s Hospital (HEC-KYJJ-2020-038-02), The trial was registered at www.chictr.org.cn (ChiCTR2100053006).

## Background

Opioids, including fentanyl and sufentanil, are widely used for the induction of anaesthesia because of their rapid onset, better analgesic effects, and favourable effect on cardiovascular stability. However, the administration of sufentanil can elicit cough, which is severe enough to result in morbidity [1]. Explosive coughs may increase intra-cranial, intraocular, and intra-abdominal pressure, and in turn, can give rise to cerebral aneurysm, cerebral trauma, cerebral hernia, and reactive airway disease [1, 2]. Moreover, explosive spasmodic coughing has been reported to cause massive engorgement of the tongue and hypopharynx, which can lead to acute airway obstruction and severe hypoxia in the paediatric population [3].

Lidocaine is an intravenously administered local anaesthetic commonly used as an adjuvant for general anaesthesia. Intravenous lidocaine has been proposed as a prophylactic to supress cough in children, even in doses as low as 0.5 mg/kg [4]. We hypothesise that intravenous lidocaine reduces sufentanil-induced cough (SIC) in paediatric patients in a dose-dependent manner. The selection of SIC as the primary outcome was guided by the need for clinically meaningful outcomes with potential benefits.

## Methods

### Study design

This manuscript adhered to the applicable CONSORT guidelines. This was a single-centre, parallel-group, randomised, double-blind, controlled trial. The study was approved by the Institutional Review Board of Yichang Central People’s Hospital, No.183 Yiling Avenue, Wujiagang District, Yichang City, Hubei Province, China (HEC-KYJJ-2020-038-02), and written informed consent was obtained from the parents or guardians of each child participating in the study. The trial was registered prior to patient enrolment at chictr.org.cn (ChiCTR2100053006, Principal investigator: Yuan Gong, http://www.chictr.org.cn/showproj.aspx?proj=136605, Date of registration: 8 November 2021). The study was conducted from 1 December 2021 to 30 May 2022 at Yichang Central People’s Hospital.

### Patients

Patients aged 3–12 years (American Society of Anesthesiologists physical status class I–II) who were scheduled for elective tonsillectomy with or without adenoidectomy were enrolled. The patients were randomised to each group in an equal ratio. Randomisation was computer generated, and every patient was assigned a patient code. The children were divided into four groups according to the dose of lidocaine they would receive: Group A (0 mg.kg-1), Group B (1 mg.kg-1), Group C (1.5 mg.kg-1), and Group D (2 mg.kg-1). The exclusion criteria were as follows: chronic cough, a history of steroid or bronchodilator treatment, reactive airway disease, upper airway infection 2 weeks prior to the procedure, therapy with angiotensin-converting enzyme inhibitors, gastro-oesophageal reflux, morbid obesity, known allergy to any of the study drugs, use of medications or nutraceuticals known to affect blood pressure (BP) and heart rate (HR), an operation lasting > 2 h, and the need for more than two intubation attempts.

### Perioperative anaesthetic care

Preoperatively, all children fasted for 6 h and were restricted to oral intake of clear fluids for 2 h. The children entered the operating room accompanied by their parents to alleviate separation anxiety. Non-invasive BP, HR, and pulse oxygen saturation were measured, and electrocardiography was performed using a multifunction monitor (GE Healthcare, Chicago, IL, USA). The width of the BP cuff for each patient was approximately two-thirds of the upper arm length. After stabilisation for 5 min, baseline HR, systolic BP, diastolic BP, and mean arterial pressure (MAP) values were obtained from the average of three measurements performed 2 min apart. A 22-gauge i.v. catheter was subsequently inserted into a vein on the back of the hand.

After preoxygenation, the research drug of interest (lidocaine [Anhui Changjiang Pharmaceutical Co. Ltd., Wuhu city, China] or 0.9% saline) was administered via intravenous injection over a 5 min period. An anaesthesia nurse who was blind to the study prepared the drug and activated infusion pump. General anaesthesia was then induced 2 min after administration of lidocaine with the following induction protocol: sufentanil (0.25 µg.kg-1), propofol (2.0 mg.kg-1), and rocuronium (0.6 mg.kg-1). An anaesthetist who was not involved in the study graded the cough response during the injection process. When the eyelash reflex disappeared, the patient’s lungs were ventilated via a facemask with 100% oxygen. A cuffed tracheal tube was used, the size of which was selected based on a widely used formula (3.5 + age in years / 4). If any difficulty was encountered while performing facemask ventilation, the patient was excluded from the study. After intubation, the patients were administered rocuronium to maintain muscle relaxation. Anaesthesia was maintained with 2–3% sevoflurane and 50% oxygen. Haemodynamic parameters of patients, including MAP and HR, were recorded at 6 time points: 0 min at endotracheal intubation (T0), 1 min after endotracheal intubation (T1), 2 min after endotracheal intubation (T2), 3 min after endotracheal intubation (T3), 4 min after endotracheal intubation (T4), and 5 min after endotracheal intubation (T5).

At the end of the operation, sevoflurane was discontinued, and neostigmine (0.04 mg.kg-1) and atropine (0.02 mg.kg-1) were administered to antagonise any residual neuromuscular blockade. Following the completion of the surgery, oral suction was performed immediately with the patient still under anaesthesia. Extubation was performed while the patient was recovering after confirming an adequate tidal volume, a regular spontaneous respiratory pattern, and purposeful behaviour (i.e., eyes opened on request). After extubation, an anaesthetist who was not involved in the study assessed recovery from anaesthesia, scored the throat pain, and graded the cough response. The patients were then monitored for at least 5 min with 100% oxygen via a facemask to allow regular spontaneous respiration, and they were subsequently transferred to post-anaesthesia care units (PACUs) after extubation. Time to extubation (time from sevoflurane discontinuation to tracheal extubation) and the cough response grade during extubation were recorded. Electrocardiography, peripheral pulse oximetry, and non-invasive BP measurements were also performed.

A patient was discharged from the PACU when their Steward score was higher than 4. Other postoperative care was performed according to the practices of local clinicians.

### Primary outcome

The primary outcome was the grade of SIC during induction of general anaesthesia. The grade of cough was assessed using a 4-point scale (none = 0; mild = 1; moderate = 2; and severe ≥ 3) [5].

### Secondary outcomes

The secondary outcomes were the incidence of SIC, MAP, and HR at T0, T1, T2, T3, T4, and T5. Additionally, haemodynamic instability was defined as changes of ± 20% from baseline, and bradycardia was defined as changes of -20% from baseline. Safety elements included cardiac events during surgery (arrhythmia, bradycardia) and rescue treatment.

### Statistical analysis

The sample size was calculated based on an expected difference of SIC grade of 1 from a preliminary experiment with 80% power (α = 0.05, β = 0.2), which indicated that 47 patients were required per group. Patient characteristics (including age, height, and weight); incidences of laryngospasm, bronchospasm, and stridor; and duration of surgery were expressed as the mean ± standard deviation and analysed using analysis of variance. A P-value of < 0.05 indicated a significant difference. The ED50 values of lidocaine were calculated using nonlinear regression. GraphPad Prism 8.0.2 (GraphPad Software Inc., San Diego, California, USA) was used for all analyses.

## Results

### Patients

Between 1 December 2021 and 30 May 2022, 188 patients were included in the analysis and divided into four groups based on the dose of lidocaine administered. There were no significant differences in patient characteristics and time of operation between the groups (Table 1).

**Table 1:** No significant difference in patient characteristics and other operative data between the groups

	Group A (*n* = 47)	Group B (*n* = 47)	Group C (*n* = 47)	Group D (*n* = 47)
Age (years)	5.55 ± 2.02	6.02 ± 2.35	5.77 ± 1.91	5.98 ± 1.67
Height (cm)	118.34 ± 14.21	120.60 ± 16.02	117.81 ± 13.65	120.49 ± 12.02
Weight (kg)	22.88 ± 7.60	23.57 ± 8.57	22.57 ± 6.65	25.03 ± 7.22
BMI(kg/m^2^)	15.96 ± 2.65	15.75 ± 2.22	16.04 ± 2.27	16.97 ± 2.75
Sex (M⁄ F)	26/21	27/20	29/18	29/18
ASA(I/II)	47/0	47/0	47/0	47/0
time of operation(min)	29.74 ± 11.14	29.40 ± 11.10	29.43 ± 13.68	27.34 ± 11.95
time of extubation(min)	11.55 ± 3.32	13.72 ± 3.30^**†**^	13.70 ± 3.59^**†**^	13.68 ± 3.58^**†**^
incidence of laryngospasm	0	0	0	0
incidence of bronchospasm	0	0	0	0
incidence of stridor	0	0	0	0
tonsillectomy with or without adenoidectomy	45/2	47/0	47/0	46/1

Values are mean ± standard deviation or numbers. †*P* < 0.05 vs. Group A

Group A, Lidocaine 0 mg/kg IV; Group B, Lidocaine 1 mg/kg IV; Group C, Lidocaine 1.5 mg/kg IV; Group D, Lidocaine 2 mg/kg IV

### Primary outcomes

**Fig. 1:**
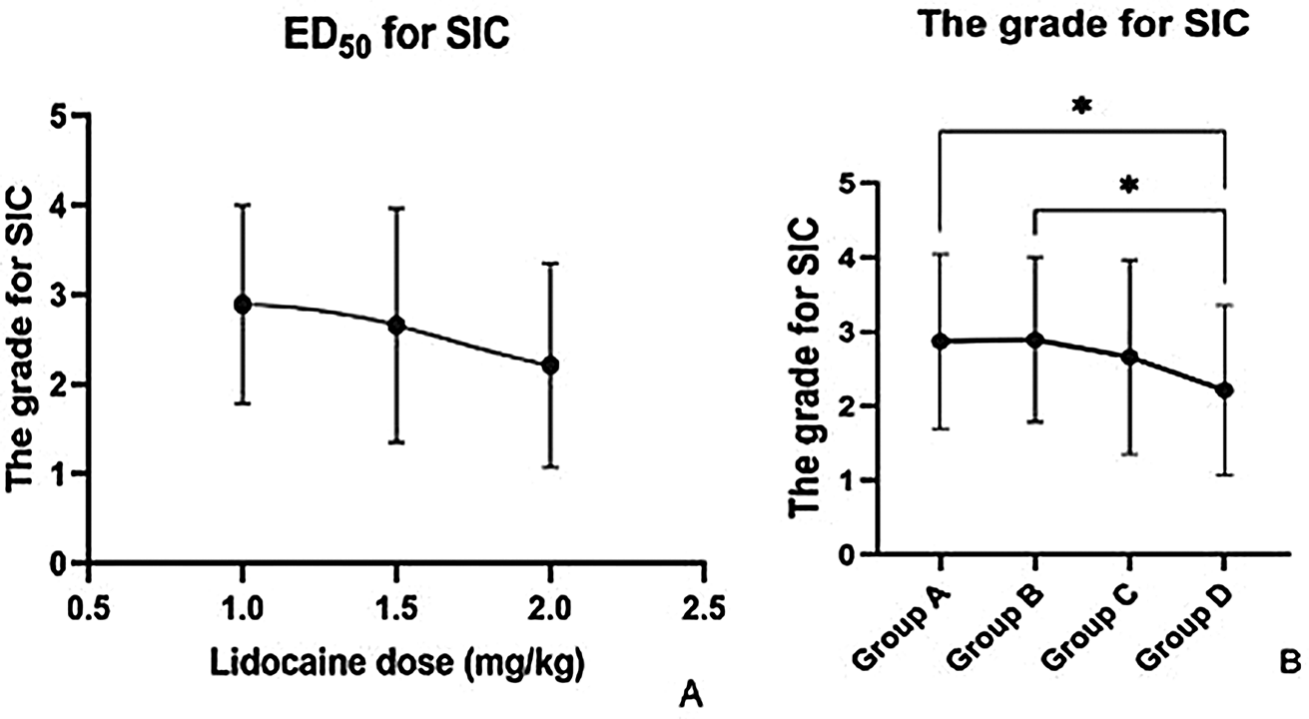
Lidocaine reduces the incidence of sufentanil-induced cough in a dose-dependent manner

Lidocaine reduces the grade of SIC in a dose-dependent manner, and the ED_50_ of intravenous lidocaine for SIC was 1.75 mg/kg (Fig. 1A). The grade of SIC in group D was lower than that in groups A and B. In group A, 20 patients (43%) had severe cough and 10 (21%) had moderate cough, whereas in group D, nine patients (19%) had moderate and nine (19%) had severe cough. The differences between groups A and D (*P* = 0.04) and between groups B and D (*P* = 0.03) were statistically significant (Fig. 1B).

### Secondary outcomes

The incidence of SIC in groups A, B, C, and D was 81%, 87%, 68%, and 64%, respectively, and the difference between groups B and C (*P* = 0.03) and between groups B and D (*P* = 0.0083) was statistically significant. There was no statistical difference in haemodynamic parameters between groups, and the incidence of severe cough was statistically different group D and group A (*P* < 0.0001), between group D and group B (*P* < 0.0001), and between group D and group C (*P* < 0.0001) respectively.There was a significant difference in the time of extubation between groups, with approximately 2 min longer required in groups B, C and D than in group A (*P* < 0.05). Our trial showed that intravenous lidocaine has no effect on haemodynamic stability. No perioperative laryngospasm, bronchospasm, or stridor was observed in any of the patient groups; Moreover, no severe complications, such as arrhythmia, were found in any of the groups.

## Discussion

SIC is a drug-related complication which is rarely noticed because it is transient. Previous studies have demonstrated that intravenous lidocaine can reduce fentanyl-induced cough in adult patients [6, 7]. Because the cause of cough in children differs from that in adults, the treatment approaches also differ. Our trial demonstrated that intravenous lidocaine reduces the grade and incidence of SIC in children. Additionally, we first demonstrated that lidocaine supresses SIC in a dose-dependent manner, and the ED_50_ of lidocaine is 1.75 mg/kg. The underlying mechanism for lidocaine suppression of cough is still unclear. Healthy children without respiratory illnesses may experience approximately 34 cough episodes in a 24-hour period [8]. The most commonly proposed mechanisms are the suppression of the excitation of airway sensory C fibres [9], selective depression of pain transmission in the spinal cord [10], and reduction in tonic neural discharge of active peripheral nerve fibres [11].

In our study, we further found that the incidence of SIC with the administration of sufentanil in children without prophylactic treatment is up to 80%. There are three reasons for this. First, ageing reduces the risk of cough, and our patients are younger than 15 years old [12]. Second, tonsillectomy in patients with tonsillar enlargement is a cause of cough [13]. Third, the administration of opioids is another risk factor for cough. With the administration of lidocaine, the incidence of SIC was reduced, and there was a statistically significant difference between groups B and C (*P* = 0.03) and groups B and D (*P* = 0.0083).

The inappropriate response of haemodynamic parameters to endotracheal intubation can increase perioperative and postoperative morbidity and mortality [14]. Additionally, there are contradicting findings about the effect of lidocaine on haemodynamic instability [15, 16]. Our study found that intravenous lidocaine did not disturb haemodynamic stability in paediatric patients, and no severe side effects were seen. The time of extubation was longer in the intervention groups (groups B, C, and D) than in group A, with a difference of approximately 2 min. This may be attributed to the analgesic efficacy of lidocaine. Prolonged time to extubation following general anaesthesia is defined as a time of at least 15 min [17]. In our study, the time of extubation in all the groups was lesser than 15 min. Hence, we believe that the longer time to extubation in our study groups may be clinically insignificant.

There are several limitations in our study. First, this was a single-centre study, which may reduce its reliability. Second, we only included patients who underwent tonsillectomy with or without adenoidectomy, which reduces the applicability of our findings to other surgery types. Third, we only enrolled patients aged 3 to 12 years; the ED 50 values of lidocaine in patients aged > 12 years or < 3 years remain poorly understood and may reduce the applicability of lidocaine in this patient group. Fourth, we only selected 0.25 µg.kg^− 1^ as the dose of sufentanil; hence, its efficacy at higher concentrations remains uncertain.

## Conclusions

In summary, intravenous lidocaine prevented SIC in a dose-dependent manner, and the ED_50_ of lidocaine was 1.75 mg/kg. Without prophylactic treatment, the rate of SIC was 80% in our study. No severe side effects were seen during our trial.

## Data Availability

The data associated with the paper are available from the corresponding author (YG) on reasonable request.

## Declarations

### Clinical trial number and registry URL

The study was approved by the institutional review board of Yichang Central People’s Hospital (HEC-KYJJ-2020-038-02). The trial was registered on 08 November, 2021, at www.chictr.org.cn (ChiCTR2100053006). The study was conducted from 01 December, 2021, to 30 May 2022, at Yichang Central People’s Hospital by Hu Yang, Ming-cheng Du, Guo-hong Zhu, Xiang Long, Jing-jing Jiang, and Yuan Gong.

## Abbreviations

SIC: Sufentanil-induced cough
BP: Blood pressure
HR: Heart rate
MAP: Mean arterial pressure
PACUs: Post-anaesthesia care units
